# Anxiety symptoms in older Chinese adults in primary care settings: Prevalence and correlates

**DOI:** 10.3389/fpubh.2022.1009226

**Published:** 2022-10-04

**Authors:** Qin Xie, Yan-Min Xu, Bao-Liang Zhong

**Affiliations:** ^1^Department of Psychiatry, Wuhan Mental Health Center, Wuhan, China; ^2^Department of Clinical Psychology, Wuhan Hospital for Psychotherapy, Wuhan, China

**Keywords:** older adults, primary care, anxiety symptoms, cross-sectional survey, China

## Abstract

**Background:**

Integrating mental health services into primary care is a potentially cost-effective way to decrease the treatment gap for anxiety in older adults but data on the epidemiology of anxiety symptoms in older Chinese adults in primary care settings have been very limited. This study investigated the prevalence and correlates of anxiety symptoms in Chinese older primary care patients.

**Methods:**

A total of 753 older primary care patients (≥65 years) were consecutively recruited from 13 primary care clinics in Wuhan, China, and interviewed with the validated Chinese version of the short form of the Geriatric Anxiety Inventory (GAI-SF).

**Results:**

The prevalence of anxiety symptoms (GAI-SF ≥ 3) in older primary care patients was 21.1%. Statistically significant correlates of anxiety symptoms were female sex (vs. male, OR = 1.85, *P* = 0.002), poor economic status (vs. good, OR = 2.31, *P* = 0.013), fair and poor family relationship (vs. good, OR = 1.85, *P* = 0.006), hypertension (OR = 2.01, *P* < 0.001), chronic gastric ulcer (OR = 6.82, *P* < 0.001), and Parkinson's disease (OR = 7.83, *P* = 0.031).

**Conclusions:**

Anxiety symptoms are prevalent among older adults attending primary care clinics. Efforts for preventing or reducing anxiety symptoms in older primary care patients may be more useful to target those who are women, have poor financial status, don't have a good family relationship, suffer from hypertension, have chronic gastric ulcer, and suffer from Parkinson's disease.

## Introduction

Anxiety symptoms and disorders are common among the elderly population and have been associated with increased risk of comorbidity with depression (1), major medical conditions (1, 2), cognitive impairment (1), poor quality of life (1), delayed recovery from illnesses (1, 2), severe disability (3), and mortality in older adults (1–3). In China, results from a meta-analysis of population-based studies show that as high as 22.1 and 6.8% of the older adults suffer from anxiety symptoms and disorders, respectively (4). Nevertheless, late-life anxiety is often underdiagnosed, misdiagnosed, and inappropriately treated in both community and clinical settings and its treatment gap is very large in both China and worldwide (5–9). Compared to the prevailing research focus on late-life depression and cognitive disorders, empirical clinical studies on prevalence, clinical characteristics, treatment, and management of anxiety in older adults are very limited (2, 10–13).

Given the wide availability and convenient accessibility of primary care services in communities and older adults' preferences for seeking treatment in primary care settings in China (14–16), integrating mental health services into primary care is a potentially cost-effective way to decrease the treatment gap for anxiety and other common mental health problems in older adults (17–19). To facilitate the planning and provision of old age mental health services, a detailed knowledge about the clinical epidemiology of late-life anxiety in Chinese primary care settings is urgently warranted. To our knowledge, in mainland China, only two studies have examined anxiety symptoms in older patients at urban primary care clinics in Shanghai, China (one in 2016 and the other in 2017) (20, 21). Both studies used the Generalized Anxiety Disorder 7-item (GAD-7) scale to assess the presence of anxiety symptoms in convenience samples of older primary care attenders and found that prevalence rates of anxiety symptoms were 13.5 and 17.2%, respectively. Factors significantly associated with anxiety symptoms in older primary care patients included younger age, female sex, educational attainment of senior middle school (vs. junior), physical deterioration, major medical conditions, physical inactivity, high economic burden due to medical care spending, inadequate family support, and inadequate peer support.

However, since GAD-7 scale is developed to screen for GAD only (22), strictly speaking, findings from the two studies only denote epidemiological characteristics of GAD symptoms in older adults receiving primary care. In fact, detecting anxiety symptoms in older adults is particularly challenging because, in comparison to young and middle-aged adults, older adults have different symptom presentations of anxiety (i.e., fear of being a burden on their families), they are more likely to minimize or deny their own anxiety symptoms, and their anxiety symptoms are more likely to be confused with somatic symptoms and cognitive impairment (23–25). Therefore, importantly, because of the unique clinical features of anxiety in later life (1, 6), the assessment results of anxiety symptoms by using a generic anxiety scale such as GAD-7, which is not specific to the elderly population, are potentially problematic. Further, another significant limitation of the above-mentioned studies is their poor sample representativeness because no rural older primary care patients were included and the participants were recruited conveniently. So the epidemiological characteristics of late-life anxiety symptoms in the elderly primary care population in China remain unknown. In addition, there is evidence that prevalence of anxiety symptoms differs across major medical conditions (26) but the two prior studies only reported the significant associations between anxiety symptoms and physical deterioration and major medical conditions, so it remains unknown which major medical condition contributes to the risk of anxiety symptoms in older adults who visit primary care physicians (PCPs) for their physical conditions.

In western countries, the recognition and management of anxiety and other common mental problems among older adults presenting to primary care are also of great clinical concern but the clinical characteristics of anxiety still have not been well-characterized (27, 28). There have been many studies investigating the prevalence of anxiety in elderly primary care attenders in Spain, United States, and other western countries, and rates of anxiety symptoms and disorders were reported to be 15.7–26.9% and 6.3–22.3%, respectively (29–37). However, because anxiety was not the primary outcome of interest of these available studies, only two reported significant correlates of anxiety symptoms, including female sex, marital status of “unmarried,” living alone, life stress events, and medical comorbidity (33, 35). Similar to the above-reviewed studies in mainland China, no detailed information on the association of anxiety symptoms with a specific medical comorbidity was provided. Further, these available studies used a variety of instruments to assess anxiety symptoms, including Goldberg Anxiety and Depression Scale, Hospital Anxiety and Depression Scale, the State-Trait Anxiety Inventory, and GAD-7 scale (33–36), but none of which used instruments that were specific to late-life anxiety.

To fill these knowledge gaps, this study investigated the prevalence and correlates of anxiety symptoms in older adults receiving primary care in Wuhan, the largest municipality in central China with a population of more than 10 million (38), with a particular focus on association between anxiety symptoms and a specific type of major medical condition. Like many other large municipalities in China, the population in Wuhan is aging rapidly in recent years and it had become an aged society since 2018 (39). The rapid aging has posed significant challenges to the healthcare system in Wuhan, including the mental healthcare for its elderly population. To help address the unmet mental health needs of the elderly population in Wuhan and other major municipalities in primary care settings, a first necessary step is to examine the epidemiological characteristics mental health problems among older adults receiving primary care.

## Methods

### Sampling and participants

Between October 2015 and November 2016, a large-scale multi-center cross-sectional survey was conducted in seven urban and six rural primary care clinics in Wuhan, China (14, 17, 40, 41). The purpose of this survey was to investigate the clinical epidemiology of a variety of mental health problems in older primary care patients, including depression, anxiety, suicidal ideation, and quality of life. The current study focused on anxiety symptoms of the primary care patients. Details of the sampling have been described in published articles (14, 17, 40, 41). We consecutively included patients who were 65 years old or older, saw PCPs in the 13 selected primary care clinics during the survey period, and were willing to join the study. We excluded patients who cannot complete the interview due to very severe physical illnesses, too severe cognitive impairment, and psychotic symptoms.

In our pilot study, the prevalence of anxiety symptoms was 30%. Accordingly, we set parameters for the sample size estimation as below: the 30% prevalence, a 0.08 confidence interval (CI) width, a two-sided 0.05 type I error rate, and an 80% response rate. The required minimum sample size was estimated to be 660 by using the formula for sample size estimation for cross-sectional studies (42).

Prior to the formal study, the survey protocol was approved by the Institutional Review Board of Wuhan Mental Health Center (approval number: WMHC-IRB-S065). All participants and their guardians (if necessary) provided informed consent before the interview.

### Instruments and procedures

All participants were interviewed by 26 PCPs with a questionnaire that was specifically developed for this survey. Interviewees were older adults and their caregivers (when present). The 26 PCPs had been trained and were deemed to be qualified to administer the questionnaire. Before the main study, a pilot study with a convenient sample of 50 older primary healthcare patients was carried out to test the feasibility of the intended procedures and the questionnaire. The survey questionnaire was revised and finalized after the pilot study.

The candidate correlates to be examined included sociodemographics, life style, and major medical conditions. Sociodemographic factors in the questionnaire were sex, age, education level, marital status, self-rated economic status, type of main jobs held by older adults during their young and middle-aged years (mental vs. physical labor), urban and rural residence place, living alone, and self-rated relationship with family members (17).

Lifestyle factors included currently smoking and habit of regular physical exercise (43, 44). A checklist was used to assess the presence of 12 major medical conditions: hypertension, diabetes, heart disease, stroke and other cerebrovascular diseases, chronic obstructive pulmonary disease (COPD), cancer, tuberculosis, chronic gastric ulcer, Parkinson's disease, anemia, hepatitis cirrhosis, and arthritis (14). Diagnoses of major medical conditions were made through interviews, physical examinations, chart review, and completion of laboratory, radiology, and other tests (when necessary).

The primary outcome, anxiety symptoms, was assessed with the Chinese version of the short form of Geriatric Anxiety Inventory (GAI-SF), which is an elderly-specific scale for assessing the severity of anxiety symptoms within the past week and has five questions with a yes-no response format (45, 46). Compared to generic anxiety scales, the GAI-SF has several strengths: minimal somatic symptoms to enable the distinction of psychiatric symptoms from physical illnesses, relative brevity to reduce respondent burden, a binary and single direction response format to decrease the cognitive load of respondents, and simple wording of the questions (46–48). The total GAI-SF scores range between zero and five, with three and above denoting clinically significant anxiety symptoms in China (45). The sensitivity and specificity of the Chinese GAI-SF cut-off score of three or greater for screening for ICD-10 anxiety disorders in older patients from a general hospital were 80.4 and 75.0%, respectively (45). In this study, the Cronbach's alpha coefficient of the Chinese GAI-SF was 0.855, suggesting good internal consistency.

### Statistical analysis

Prevalence rates of anxiety symptoms among the total sample and subgroups according to sociodemographics, lifestyle, and major medical conditions were calculated. Chi-square test was used to compare rates between/across subgroups. Multiple binary logistic regression with a forward stepwise entry of all significant factors in the Chi-square test was used to identify correlates of anxiety symptoms. Odds ratios (ORs) and their 95% confidence intervals (95% CIs) were used to quantify associations between factors and anxiety symptoms. Nagelkerke R Square (*R*^2^) was calculated as the indicator for goodness-of-fit of the logistic regression model and its robustness was tested by using Hosmer-Lemeshow's goodness-of-fit test (H-L test) (49). A non–statistically significant result on H-L test indicates that the model is well calibrated, so the fit is good. The statistical significance level was set at two-sided *P* < 0.05. SPSS software version 14.0 package (SPSS Inc., Chicago, IL, USA) was used for all analyses.

## Results

### Sample characteristics

Altogether, we invited 791 older primary care patients to participate in the study and 753 agreed and completed the survey questionnaire (response rate: 95.2%). The mean age of the study sample was 72.8 years (standard deviation = 6.0, range = 65–97) and 404 (53.7%) were females. Characteristics of the survey sample and prevalence rates of anxiety symptoms among different subgroups are shown in Table 1.

**Table 1:** Characteristics of the sample of older adults in primary care settings and prevalence rates of anxiety symptoms by sample characteristics.

**Characteristics**		**No. of older adults**	**No. of older adults with anxiety symptoms**	**Prevalence (%)**	**χ^2^**	***P***
Sex	Male	349	56	16.0	10.037	0.002
	Female	404	103	25.5		
Age (years)	65–74	488	106	21.7	0.305	0.580
	75+	265	53	20.0		
Education	Illiterate	174	50	28.7	8.131	0.017
	Primary school	216	43	19.9		
	Middle school and above	363	66	18.2		
Marital status	Married	526	114	21.7	0.326	0.568
	Others^*^	227	45	19.8		
Self-rated financial status	Good	132	26	19.7	10.080	0.006
	Fair	533	103	19.3		
	Poor	88	30	34.1		
Main occupation before older adulthood	Mental labor	220	35	15.9	5.058	0.025
	Manual labor	533	124	23.3		
Residence place	Urban	408	87	21.3	0.023	0.879
	Rural	345	72	20.9		
Living alone	No	670	146	21.8	1.665	0.197
	Yes	83	13	15.7		
Self-rated family relationship	Good	594	114	19.2	6.249	0.012
	Fair and poor^**^	159	45	28.3		
Currently smoking	No	633	140	22.1	2.391	0.122
	Yes	120	19	15.8		
Regular physical excise	No	319	73	22.9	1.039	0.308
	Yes	434	86	19.8		
Hypertension	No	387	63	16.3	11.181	0.001
	Yes	366	96	26.2		
Diabetes	No	638	128	20.1	2.780	0.095
	Yes	115	31	27.0		
Heart disease	No	675	135	20.0	4.868	0.027
	Yes	78	24	30.8		
Stroke and other cerebrovascular diseases	No	689	136	19.7	9.225	0.002
	Yes	64	23	35.9		
Chronic obstructive pulmonary disease	No	709	149	21.0	0.073	0.787
	Yes	44	10	22.7		
Cancer	No	748	158	21.1	0.004	0.951
	Yes	5	1	20.0		
Tuberculosis	No	751	159	21.2	0.537	0.464
	Yes	2	0	0.0		
Chronic gastric ulcer	No	728	145	19.9	18.892	<0.001
	Yes	25	14	56.0		
Parkinson's disease	No	748	156	20.9	4.569	0.033
	Yes	5	3	60.0		
Anemia	No	745	156	20.9	1.303	0.254
	Yes	8	3	37.5		
Hepatitis cirrhosis	No	751	158	21.0	1.004	0.316
	Yes	2	1	50.0		
Arthritis	No	697	143	20.5	2.019	0.155
	Yes	56	16	28.6		

* “*Others” included never married, separated, divorced, widowed, cohabitating, and remarried*.

** *Because of the very small numbers of the category of “poor” relationship (n<10), “poor” and “fair” were merged into one category*.

### Prevalence of anxiety symptoms

In total, 159 older adults were screened positive for anxiety symptoms and the corresponding 1-week prevalence of anxiety symptoms was 21.1%. Women had statistically significant higher rates of anxiety symptoms than men (25.5 vs. 16.0%, *P* = 0.002). Similar rates of anxiety symptoms were found between urban and rural older adults (21.3 vs. 20.9%, *P* = 0.879).

### Correlates of anxiety symptoms

Results from Chi-square test (Table 1) display that significantly higher rates of anxiety symptoms were observed in women (vs. men), in illiterate participants (vs. primary school or middle school and above), in participants with poor financial status (vs. good or fair), in participants who engaged in physical labor (vs. mental labor) before the older adulthood, in participants with fair and poor family relationship (vs. good), in participants with hypertension, in participants with heart disease, in participants with stroke and other cerebrovascular diseases, in participants with chronic gastric ulcer, and in participants with Parkinson's disease.

In multiple logistic regression analysis, six significant correlates of anxiety symptoms were identified (Table 2): Female sex (vs. male, OR = 1.85, *P* = 0.002), poor economic status (vs. good, OR = 2.31, *P* = 0.013), fair and poor family relationship (vs. good, OR = 1.85, *P* = 0.006), hypertension (OR = 2.01, *P* < 0.001), chronic gastric ulcer (OR = 6.82, *P* < 0.001), and Parkinson's disease (OR = 7.83, *P* = 0.031). The results of H-L test show that the multiple logistic regression model fitted the data well (*R*^2^ = 0.122, χ^2^ = 11.800, *P* = 0.107).

**Table 2:** Correlates of anxiety symptoms in older adults in primary care settings.

**Factor**	**Risk level**	**Reference level**	**Coefficient**	**Standard error**	**χ^2^**	***P***	**OR(95%CI)**
Sex	Female	Male	0.616	0.195	10.004	0.002	1.85 (1.26, 2.71)
Self-rated economic status	Poor	Good	0.837	0.338	6.151	0.013	2.31 (1.19, 4.48)
Self-rated family relationship	Fair and poor^*^	Good	0.615	0.222	7.665	0.006	1.85 (1.20, 2.86)
Hypertension	Yes	No	0.700	0.194	13.042	< 0.001	2.01 (1.38, 2.95)
Chronic gastric ulcer	Yes	No	1.920	0.433	19.699	< 0.001	6.82 (2.92, 15.94)
Parkinson's disease	Yes	No	2.057	0.956	4.633	0.031	7.83 (1.20, 50.95)

* *Because of the very small numbers of the category of “poor” relationship (n*<*10), “poor” and “fair” were merged into one category*.

## Discussion

To the best of our knowledge, this is the first study in China that examines the epidemiological characteristics of anxiety symptoms in both urban and rural older primary care patients by using an anxiety scale specific to elderly population, GAI-SF. The main findings of this study are the 21.1% prevalence of anxiety symptoms in older adults receiving primary care and six correlates of anxiety symptoms in this patient population: female sex, poor economic status, fair and poor family relationship, hypertension, chronic gastric ulcer, and Parkinson's disease.

In a community-based survey in Beijing, China, Tang and Wang used GAI to assess the presence of anxiety symptoms in older adults and found that 7.4% of them suffered from anxiety symptoms (50). Compared to this prevalence estimate in community-dwelling older adults, we found a much higher prevalence of anxiety symptoms in older primary care patients. The 21.1% prevalence of anxiety symptoms in older adults in primary care settings is similar to the 22.1% prevalence of anxiety symptoms in older adults in communities from the above-mentioned meta-analysis (4). Nevertheless, we argue that this does not indicate a similar risk of anxiety symptoms in older adults between primary care settings and communities, because all the included studies of this meta-analysis used generic anxiety scales to screen for anxiety symptoms, which tends to overestimate the prevalence of anxiety symptoms in older adults (23, 24, 51).

The high risk of anxiety symptoms in Chinese older primary care attenders may be primarily explained by their prevailing physical health problems, because many major medical conditions (i.e., hypertension, diabetes, and COPD) have been associated with anxiety symptoms and disorders (52, 53). Further, the under-recognition and under-treatment of late-life anxiety in primary care settings, particularly in Chinese primary care settings (5–9, 54), would prolong the duration of the anxiety and result in persistently high level of anxiety in Chinese older primary care patients.

Our findings on the significant associations of anxiety symptoms with female sex and poor financial status are consistent with elevated risk of anxiety symptoms in older women and socioeconomically disadvantageous older adults in earlier studies (55–57). Because family members are a significant source of social support for older Chinese adults and family support plays an important role in the mental well-being of older Chinese adults (58–60), our finding on the significantly higher risk of anxiety symptoms in older adults without a good family relationship is expected. In general, rural older adults are more likely to develop anxiety symptoms than urban older adults because of their lower socioeconomic status and the inadequate health service resources in rural regions (4). However, we found similar risk of anxiety symptoms between urban and rural older adults in this study, which is not in line with prior studies (20, 21, 33, 36). Perhaps, some stronger factors (i.e., major medical conditions) may mask or affect the effect of residence place on the risk of anxiety in older primary care patients.

Accumulating evidence has shown that hypertension and peptic ulcers are psychosomatic diseases and there are reciprocal relationships between hypertension and peptic ulcers and mental health problems such as anxiety (61–64). In addition, anxiety, as a stress response, can also be resulted from some major medical conditions if they persist long enough to become chronic stressors (65). In line with these earlier studies, hypertension and chronic gastric ulcer were significantly associated with anxiety symptoms in older primary care patients. It has been well-recognized that anxiety is a common psychiatric comorbidity in Parkinson's disease and the comorbidity between anxiety and Parkinson's disease can be explained by their shared neuropathophysiology such as the loss of adrenergic and serotonergic neurons (66, 67). As a result, Parkinson's disease was found to be a significant correlate of anxiety symptoms in this study.

This study has a few limitations. First, because of the cross-sectional assessment of anxiety symptoms and associated factors, the current study cannot ascertain the causal relationships between identified correlates and anxiety symptoms. Second, additionally assessing the presence of anxiety disorders according to DSM-IV or other diagnostic criteria would increase the clinical relevance of the study findings but we did collect data on this mental health outcome. Third, we did not investigate the mental health help-seeking behaviors of older adults with anxiety symptoms, which can inform the mental health policy-making and planning in primary care settings. Fourth, our sample of older primary care attenders was recruited from primary care clinics of a large city in central China. Older patients of primary care clinics in other cities were not included, potentially limiting the generalizability of our findings. Fifth, some other factors associated with anxiety in older adults, such as personality and stressful life event, were not measured in this study. Finally, because our study was conducted before the COVID-19 pandemic and the pandemic has had a long-term and far-reaching negative impact on the mental health of older adults (19, 68), studies during the pandemic and post-pandemic periods are warranted to further examine the influence of the pandemic on the risk of anxiety and its associated factors in older primary care attenders.

In conclusion, anxiety symptoms are prevalent among older adults receiving primary care in China. Given the many negative outcomes associated with anxiety, it is necessary to integrate mental health services into the routine primary care services. To achieve the goal of early detection and timely treatment, health services for older primary care patients need to include periodic assessment of anxiety symptoms, psychosocial support to improve the mental well-being, and, if necessary, psychiatric referral and treatment. Efforts for preventing or reducing anxiety symptoms in older primary care patients may be more useful to target those who are women, have poor financial status, don't have a good family relationship, suffer from hypertension, have chronic gastric ulcer, and suffer from Parkinson's disease.

Our findings on some correlates of anxiety symptoms are clinically relevant because family relationship, hypertension, chronic gastric ulcer, and Parkinson's disease are potentially modifiable or treatable. The significant associations of anxiety symptoms with these social and medical problems further indicate the clinical needs for collaborative multidisciplinary management services for reducing the burden of anxiety in older primary care patients, which should integrate social work outreach services to promote family relationship, mental health services to relive anxiety symptoms, and primary care services to manage hypertension, chronic gastric ulcer, and Parkinson's disease.

## Data availability statement

The original contributions presented in the study are included in the article/supplementary material, further inquiries can be directed to the corresponding author.

## Ethics statement

The studies involving human participants were reviewed and approved by the Institutional Review Board of Wuhan Mental Health Center. The patients/participants provided their written informed consent to participate in this study.

## Author contributions

QX: acquisition and analysis of data for the study, drafting the paper, and interpretation of data for the study. QX and Y-MX: design and acquisition of data for the study. B-LZ: drafting the paper, revising the paper for important intellectual content, and interpretation of data for the study. All authors contributed to the article and approved the submitted version.

## Funding

This work was supported by National Natural Science Foundation of China (Grant Number: 71774060), 2015 Irma and Paul Milstein Program for Senior Health Awards from the Milstein Medical Asian American Partnership Foundation, the Young Top Talent Programme in Public Health from Health Commission of Hubei Province (PI: B-LZ), and Wuhan Health and Family Planning Commission (Grant Number: WX17Q30; WG16A02; WG14C24). The funding source listed had no role in study design; in the collection, analysis and interpretation of data; in the writing of the report; and in the decision to submit the paper for publication.

## Conflict of interest

The authors declare that the research was conducted in the absence of any commercial or financial relationships that could be construed as a potential conflict of interest.

## Publisher's note

All claims expressed in this article are solely those of the authors and do not necessarily represent those of their affiliated organizations, or those of the publisher, the editors and the reviewers. Any product that may be evaluated in this article, or claim that may be made by its manufacturer, is not guaranteed or endorsed by the publisher.
